# COVID-19 Vaccination Coverage and Vaccine Confidence by Sexual Orientation and Gender Identity — United States, August 29–October 30, 2021

**DOI:** 10.15585/mmwr.mm7105a3

**Published:** 2022-02-04

**Authors:** A.D. McNaghten, Noel T. Brewer, Mei-Chuan Hung, Peng-Jun Lu, Demetre Daskalakis, Neetu Abad, Jennifer Kriss, Carla Black, Elisabeth Wilhelm, James T. Lee, Adi Gundlapalli, Janet Cleveland, Laurie Elam-Evans, Kimberly Bonner, James Singleton

**Affiliations:** ^1^CDC COVID-19 Emergency Response Team; ^2^University of North Carolina, Chapel Hill, North Carolina; ^3^Leidos Inc, Reston, Virginia.

Lesbian, gay, bisexual, and transgender (LGBT) populations have higher prevalences of health conditions associated with severe COVID-19 illness compared with non-LGBT populations (*1*). The potential for low vaccine confidence and coverage among LGBT populations is of concern because these persons historically experience challenges accessing, trusting, and receiving health care services (*2*). Data on COVID-19 vaccination among LGBT persons are limited, in part because of the lack of routine data collection on sexual orientation and gender identity at the national and state levels. During August 29–October 30, 2021, data from the National Immunization Survey Adult COVID Module (NIS-ACM) were analyzed to assess COVID-19 vaccination coverage and confidence in COVID-19 vaccines among LGBT adults aged ≥18 years. By sexual orientation, gay or lesbian adults reported higher vaccination coverage overall (85.4%) than did heterosexual adults (76.3%). By race/ethnicity, adult gay or lesbian non-Hispanic White men (94.1%) and women (88.5%), and Hispanic men (82.5%) reported higher vaccination coverage than that reported by non-Hispanic White heterosexual men (74.2%) and women (78. 6%). Among non-Hispanic Black adults, vaccination coverage was lower among gay or lesbian women (57.9%) and bisexual women (62.1%) than among heterosexual women (75.6%). Vaccination coverage was lowest among non-Hispanic Black LGBT persons across all categories of sexual orientation and gender identity. Among gay or lesbian adults and bisexual adults, vaccination coverage was lower among women (80.5% and 74.2%, respectively) than among men (88.9% and 81.7%, respectively). By gender identity, similar percentages of adults who identified as transgender or nonbinary and those who did not identify as transgender or nonbinary were vaccinated. Gay or lesbian adults and bisexual adults were more confident than were heterosexual adults in COVID-19 vaccine safety and protection; transgender or nonbinary adults were more confident in COVID-19 vaccine protection, but not safety, than were adults who did not identify as transgender or nonbinary. To prevent serious illness and death, it is important that all persons in the United States, including those in the LGBT community, stay up to date with recommended COVID-19 vaccinations.

NIS-ACM collects data from adults aged ≥18 years using a random-digit–dialed sample of cellular telephone numbers (*3*). Data collected during August 29–October 30, 2021 from 153,062 respondents were weighted to represent the noninstitutionalized U.S. adult population and to match the number of adults who received ≥1 dose* of COVID-19 vaccine as reported by jurisdictions to CDC.^†^ The response rate was 20.9% in both September and October.^§^ Sexual orientation was assessed with the question, “What best describes your sexual orientation? Is it heterosexual or straight; lesbian or gay; bisexual; or something else?” Gender identity was assessed with the question, “Would you consider yourself as transgender or nonbinary?” Adults who answered “don’t know” or “refused” to the sexual orientation (9,586, 6.3%) or gender identity (10,539, 6.9%) questions were excluded from the analysis.

Self-reported data on COVID-19 vaccination coverage by sociodemographic characteristics, and behavioral and social drivers of vaccination were analyzed by sexual orientation and gender identity.^¶^ Assessed drivers of vaccination were concerns about COVID-19, and importance of and confidence in COVID-19 vaccines. Data were stratified by male or female sex for heterosexual, gay or lesbian, and bisexual respondents. Because persons who describe themselves as nonbinary do not identify as male or female, gender identity was not stratified by male or female sex. Analyses used t-tests and 95% CIs to detect differences in percentages between groups, using a threshold of α = 0.05 for statistical significance. Analyses were performed using SAS (version 9.4; SAS Institute) and SUDAAN (version 11.0.3; RTI International). This activity was reviewed by CDC and was conducted consistent with applicable federal law and CDC policy.**

Among 143,476 survey respondents with nonmissing responses to the sexual orientation question, 3,941 (2.7%) identified as gay or lesbian and 4,395 (3.1%) as bisexual; of the 142,523 survey respondents with nonmissing responses to the gender identity question, 5,594 (3.9%) identified as transgender or nonbinary. Receipt of ≥1 dose of a COVID-19 vaccine was higher among gay or lesbian adults (85.4%) than among heterosexual (76.3%; p<0.05) or bisexual (76.3%) adults (Table 1). Among gay or lesbian adults and bisexual adults, a higher percentage of men (88.9% and 81.7%, respectively) than women (80.5% and 74.2%, respectively) reported receiving ≥1 COVID-19 vaccine dose (Table 2). The percentage of transgender or nonbinary adults who reported receiving ≥1 dose of COVID-19 vaccine (75.7%) was statistically similar to that among adults who did not identify as transgender or nonbinary (76.7%).

**TABLE 1:** COVID–19 vaccination status and intent, by sex, gender identity, sexual orientation, and race/ethnicity — National Immunization Survey Adult COVID Module, United States, August 29–October 30, 2021

Characteristic	Unweighted no.	% (95% CI)*				
		Vaccinated (n= 131,215)		Unvaccinated (n = 21,176)		
		≥1 dose	Fully vaccinated^†^	Definitely plan to get vaccinated	Probably will get vaccinated or unsure	Probably or definitely will not get vaccinated
**Sex**						
Male (Ref.)	74,387	74.5 (73.8–75.2)	71.5 (70.7–72.2)	2.3 (2.1–2.6)	8.0 (7.5–8.4)	15.2 (14.6–15.8)
Female	77,372	78.8 (78.2–79.4)^§^	76.1 (75.5–76.7)^§^	2.0 (1.8–2.3)	7.0 (6.6–7.4)^§^	12.2 (11.7–12.7)^§^
**Transgender or nonbinary**						
No (Ref.)	136,929	76.7 (76.2–77.2)	73.9 (73.4–74.4)^§^	2.1 (2.0–2.3)	7.4 (7.1–7.7)	13.8 (13.4–14.2)
Yes	5,594	75.7 (73.3–78.0)	71.4 (68.8–73.8)	2.8 (2.0–3.8)	8.0 (6.7–9.6)	13.5 (11.7–15.5)
**Sexual orientation**						
Heterosexual/Straight (Ref.)	132,608	76.3 (75.8–76.8)	73.5 (73.0–74.0)	2.1 (1.9–2.3)	7.4 (7.1–7.7)	14.2 (13.8–14.6)
Gay or lesbian	3,941	85.4 (82.5–87.8)^§^	83.1 (80.2–85.6)^§^	1.5 (0.9–2.5)	4.9 (3.5–7.0)^§^	8.2 (6.3–10.5)^§^
Bisexual	4,395	76.3 (73.6–78.9)	72.6 (69.8–75.2)	4.1 (3.0–5.6)^§^	9.4 (7.6–11.7)	10.2 (8.6–12.1)^§^
Something else	2,532	75.3 (71.7–78.7)	71.9 (68.2–75.4)	4.2 (2.7–6.5)^§^	8.6 (6.6–11.0)	11.9 (9.5–15.0)
**Race/Ethnicity**						
White, non-Hispanic (Ref.)	96,923	76.9 (76.3–77.5)	74.5 (73.9–75.1)	1.8 (1.6–2.0)	6.4 (6.1–6.8)	14.9 (14.4–15.4)
Black, non-Hispanic	17,159	74.1 (72.7–75.5)^§^	69.7 (68.3–71.1)^§^	3.2 (2.7–3.8)^§^	10.9 (10.0–11.9)^§^	11.8 (10.7–12.9)^§^
Hispanic	19,344	76.4 (75.1–77.6)	72.5 (71.2–73.8)^§^	3.1 (2.6–3.7)^§^	9.1 (8.3–10.0)^§^	11.4 (10.5–12.4)^§^
Other, non-Hispanic	15,037	80.1 (78.6–81.6)^§^	78.0 (76.5–79.5)^§^	1.8 (1.3–2.3)	6.5 (5.6–7.5)	11.6 (10.4–12.9)^§^

**Abbreviation**: Ref. = referent group.

* Weighted estimate.

^†^ Respondents self-reported receipt of ≥2 doses of BNT162b2 (Pfizer-BioNTech), mRNA-1273 (Moderna), or other COVID-19 vaccine, or 1 dose of Ad.26.COV2.S (Janssen [Johnson & Johnson]) COVID-19 vaccine.

^§^ Compared with ref, p<0.05.

**TABLE 2:** COVID-19 vaccination (≥1 dose) coverage, by demographic characteristics stratified by sexual orientation, gender identity, and sex — National Immunization Survey AdultCOVID Module, United States, August 29–October 30, 2021

Characteristic	% (95% CI)*												
	Sexual orientation											Gender identity	
	Heterosexual/Straight			Gay or lesbian			Bisexual			Something else	Not transgender or nonbinary		Transgender or nonbinary
	Overall	Men	Women	Overall	Men	Women	Overall	Men	Women	Total			
**Total**	**76.3 (75.8–76.8)**	**73.8 (73.0–74.5)**	**78.9 (78.2–79.6)**	**85.4 (82.5–87.8)**	**88.9 (85.2–91.8)**	**80.5 (75.6–84.5)**	**76.3 (73.6–78.9)**	**81.7 (76.6–85.9)**	**74.2 (70.8–77.3)**	**75.3 (71.7–78.7)**	**76.7 (76.2–77.2)**		**75.7 (73.3–78.0)**
**Race/Ethnicity**													
White, non-Hispanic	76.5 (75.8–77.1)	74.2 (73.3–75.1)	78.6 (77.7–79.5)	91.7 (89.1–93.8)	94.1 (90.9–96.2)	88.5 (83.4–92.1)	76.4 (73.0–79.5)	81.4 (74.3–86.9)	74.6 (70.6–78.2)	78.8 (73.7–83.1)	76.9 (76.3–77.5)		77.5 (74.1–80.6)
Black, non-Hispanic	74.2 (72.7–75.6)	72.5 (70.3–74.6)	75.6 (73.6–77.4)	66.8 (54.9–76.9)	76.6 (58.3–88.5)	57.9 (41.9–72.3)	68.6 (59.6–76.3)	79.8 (62.9–90.3)	62.1 (51.2–71.9)	77.9 (68.2–85.3)	74.0 (72.5–75.4)		69.3 (63.2–74.7)
Hispanic	76.1 (74.6–77.4)	72.0 (69.9–74.0)	80.5 (78.6–82.3)	79.6 (71.7–85.8)	82.9 (71.1–90.6)	72.6 (60.3–82.3)	80.4 (74.1–85.4)	82.4 (70.8–90.0)	79.5 (71.7–85.6)	71.2 (61.9–79.0)	76.3 (74.8–77.6)		78.1 (72.5–82.8)
Other, non-Hispanic	80.3 (78.6–81.9)	76.9 (74.4–79.3)	84.4 (82.3–86.2)	80.7 (70.1–88.1)	80.1 (64.5–90.0)	81.2 (65.0–91.0)	75.6 (63.2–84.7)	83.3 (62.5–93.7)	73.0 (57.2–84.6)	74.5 (64.7–82.3)	80.6 (79.0–82.1)		74.7 (66.4–81.5)
**Household income**													
Below poverty**^†^**	64.6 (62.7–66.3)	64.6 (61.9–67.3)	64.4 (62.0–66.8)	74.3 (62.4–83.5)	77.7 (61.1–88.6)	70.2 (51.7–83.9)	60.1 (52.6–67.1)	63.4 (48.6–76.1)	58.7 (49.9–67.0)	68.0 (59.6–75.4)	63.9 (62.1–65.7)		65.3 (58.7–71.4)
Above poverty, <$75,000	74.7 (73.8–75.6)	72.0 (70.7–73.3)	77.2 (76.0–78.4)	82.9 (77.8–87.0)	87.7 (80.5–92.5)	76.0 (68.0–82.5)	77.7 (73.4–81.5)	83.9 (76.2–89.5)	75.3 (69.9–80.0)	74.5 (68.0–80.0)	74.9 (74.0–75.8)		78.2 (74.4–81.5)
Above poverty, ≥$75,000	82.7 (82.0–83.4)	79.1 (78.0–80.2)	86.8 (85.9–87.8)	94.3 (91.8–96.0)	96.3 (94.2–97.6)	91.3 (85.4–95.0)	90.3 (86.6–93.1)	94.5 (89.3–97.3)	88.8 (83.8–92.3)	85.2 (77.1–90.8)	83.5 (82.8–84.2)		80.0 (74.7–84.4)
Unknown income	73.9 (72.8–75.1)	70.8 (69.1–72.5)	76.9 (75.3–78.4)	77.4 (67.6–84.8)	78.5 (63.8–88.3)	75.1 (60.4–85.7)	71.0 (63.9–77.1)	79.3 (66.7–88.0)	67.8 (59.3–75.3)	75.7 (68.0–82.0)	74.1 (73.0–75.2)		74.8 (69.5–79.4)
**U.S. Census region**													
Northeast	84.7 (83.9–85.5)	82.2 (80.9–83.5)	87.1 (85.9–88.2)	88.5 (82.1–92.9)	95.3 (87.6–98.3)	78.4 (66.3–87.0)	84.4 (79.0–88.5)	88.6 (79.2–94.1)	82.2 (75.3–87.5)	78.8 (71.4–84.6)	84.7 (83.8–85.5)		83.7 (79.3–87.3)
Midwest	70.7 (69.4–72.0)	68.0 (66.1–69.8)	73.4 (71.6–75.2)	83.0 (74.6–89.0)	83.5 (70.4–91.5)	82.1 (70.9–89.6)	74.1 (67.1–80.1)	92.5 (85.9–96.2)	68.2 (59.7–75.7)	68.9 (59.4–77.0)	71.0 (69.8–72.3)		73.5 (67.8–78.5)
South	72.6 (71.8–73.4)	69.8 (68.6–70.9)	75.4 (74.3–76.5)	80.2 (74.9–84.6)	85.7 (79.3–90.4)	72.1 (62.7–79.8)	69.8 (64.7–74.4)	71.9 (61.5–80.4)	69.0 (63.0–74.3)	76.4 (70.8–81.1)	72.9 (72.1–73.6)		71.2 (67.1–74.9)
West	80.7 (79.6–81.7)	78.5 (77.0–80.0)	83.0 (81.5–84.4)	92.0 (87.4–95.0)	92.5 (84.7–96.5)	91.6 (85.3–95.3)	82.0 (77.5–85.8)	84.0 (74.4–90.4)	81.2 (75.8–85.6)	74.9 (65.7–82.4)	81.2 (80.2–82.2)		79.3 (73.7–84.0)
**Urbanicity** ^§^													
MSA, principal city	78.3 (77.4–79.1)	76.6 (75.3–77.8)	79.9 (78.7–81.1)	86.8 (82.8–90.0)	92.2 (88.0–95.0)	77.2 (69.1–83.7)	80.1 (76.1–83.7)	88.3 (82.0–92.6)	76.6 (71.3–81.1)	80.7 (75.0–85.4)	79.0 (78.1–79.8)		73.9 (69.5–77.8)
MSA, nonprincipal city	77.8 (77.1–78.4)	74.9 (73.9–75.9)	80.6 (79.7–81.5)	85.3 (80.5–89.1)	87.9 (80.8–92.6)	82.4 (75.0–87.9)	76.6 (72.7–80.1)	77.7 (69.0–84.5)	76.2 (71.7–80.2)	73.6 (68.3–78.3)	77.9 (77.2–78.5)		79.0 (75.7–82.0)
Non-MSA	66.6 (65.2–67.9)	63.3 (61.3–65.3)	69.9 (68.0–71.7)	77.9 (67.2–85.9)	72.9 (55.3–85.4)	84.2 (74.9–90.6)	61.6 (51.9–70.4)	73.0 (56.7–84.8)	58.1 (46.8–68.7)	64.6 (53.3–74.5)	66.4 (65.1–67.8)		68.3 (62.0–73.9)
**SVI** ^¶^													
Low SVI	79.3 (78.4–80.2)	76.9 (75.6–78.2)	81.6 (80.4–82.8)	89.3 (84.4–92.8)	92.2 (85.7–95.9)	85.7 (77.0–91.4)	79.5 (74.0–84.2)	87.4 (78.3–93.0)	76.7 (69.6–82.5)	78.7 (70.9–84.8)	79.7 (78.9–80.6)		79.3 (74.4–83.5)
Moderate SVI	77.6 (76.7–78.4)	75.2 (73.9–76.4)	80.0 (78.8–81.1)	87.2 (83.1–90.4)	93.7 (89.6–96.2)	78.5 (70.7–84.6)	75.9 (71.3–79.9)	76.9 (67.1–84.5)	75.4 (70.1–80.0)	75.2 (68.5–80.8)	77.9 (77.0–78.7)		77.9 (73.8–81.5)
High SVI	74.3 (73.3–75.2)	71.9 (70.5–73.3)	76.5 (75.2–77.8)	83.7 (77.5–88.5)	86.3 (78.1–91.7)	79.2 (68.2–87.1)	75.8 (70.7–80.3)	85.6 (77.9–90.9)	72.3 (66.0–77.9)	73.9 (66.6–80.0)	74.7 (73.7–75.6)		74.2 (69.5–78.3)

**Abbreviations**: MSA = metropolitan statistical area; SVI = Social Vulnerability Index.

* Weighted estimate.

^†^ Poverty was derived based on the number of persons reported in the household, the reported household income, and the 2020 U.S. Census poverty thresholds.

^§^ Urbanicity status was derived based on the centroid of the zip code of residence, categorized as MSA principal city, MSA non-principal city, or non-MSA.

^¶^ SVI was categorized as low, moderate, or high based on county of residence using tertiles of SVI score as cut-points. https://www.atsdr.cdc.gov/placeandhealth/svi/index.html

Among non-Hispanic White adults, the percentage who reported receiving ≥1 COVID-19 vaccine dose was higher among gay or lesbian adults (91.7%) than among heterosexual adults (76.5%), higher among gay men (94.1%) and bisexual men (81.4%) than among heterosexual men (74.2%), and higher among gay or lesbian women (88.5%) than among heterosexual women (78.6%) (all p<0.05). The percentage of non-Hispanic White bisexual women who reported receiving ≥1 COVID-19 vaccine dose (74.6%) was lower than that among heterosexual women (p<0.05). Among Hispanic adults, the percentage who reported receiving ≥1 COVID-19 vaccine dose was higher among gay men (82.9%) than among heterosexual men (72.0%; p<0.05). Among non-Hispanic Black adults, coverage was lower among gay or lesbian women (57.9%) and bisexual women (62.1%) than among heterosexual women (75.6%) (p<0.05). Receipt of ≥1 COVID-19 vaccine dose was highest among non-Hispanic White gay men (94.1%) and lowest among non-Hispanic Black gay or lesbian women (57.9%). There were no statistically significant differences by race/ethnicity among adults who identified as transgender or nonbinary compared with those who did not identify as transgender or nonbinary.

By urbanicity,^††^ among adults residing in a Metropolitan Statistical Area (MSA) principal city, ≥1-dose vaccination coverage was higher among gay men (92.2%) and bisexual men (88.3%) than among heterosexual men (76.6%); a lower percentage of persons who identified as transgender or nonbinary (73.9%) reported receiving ≥1 dose compared with persons who did not identify as transgender or nonbinary (79.0%) (all p<0.05). Among adults residing in an MSA nonprincipal city, the percentage of gay men who reported receiving ≥1 dose (87.9%) was higher than that among heterosexual men (74.9%); among bisexual women, ≥1-dose coverage (76.2%) was lower than that among heterosexual women (80.6%) (all p<0.05). For adults living in a non-MSA, coverage was higher among gay or lesbian women (84.2%) and lower among bisexual women (58.1%) than among heterosexual women (69.9%) (all p<0.05).

Among both vaccinated and unvaccinated respondents, a higher percentage of gay or lesbian adults and bisexual adults reported they were very or moderately concerned about COVID-19 (56.8% and 51.3%, respectively) than were heterosexual adults (48.1%) (all p<0.05) (Table 3). Higher percentages of gay and bisexual men reported they were completely confident or very confident in vaccine safety (82.4% and 76.3%, respectively) than were heterosexual men (63.2%), as were bisexual women (68.1%) compared with heterosexual women (64.5%) (all p<0.05). Higher percentages of gay or lesbian adults and bisexual adults reported that they thought COVID-19 vaccine was very or somewhat important to protect oneself (90.8% and 86.8%, respectively) compared with heterosexual adults (80.4%), and higher percentages of adults who identified as transgender or nonbinary reported they thought COVID-19 vaccine was very or somewhat important to protect oneself (83.2%) compared with those who did not identify as transgender or nonbinary (80.7%) (all p<0.05).

**TABLE 3:** Behavioral and social drivers of COVID-19 vaccination, by sexual orientation and gender identity — National Immunization Survey Adult COVID Module, United States, August 29–October 30, 2021

Central attitudes and experiences	% (95% CI)*						
	Overall	Sexual orientation				Gender identity	
		Heterosexual/ Straight	Gay or lesbian	Bisexual	Something else	Not transgender or nonbinary	Transgender or nonbinary
**Total**	**153,062**	**132,608**	**3,941**	**4,395**	**2,532**	**136,929**	**5,594**
**Concerned about COVID–19 (very or moderately)**	48.5 (48.0–49.0)	48.1 (47.6–48.7)	56.8 (53.5–60.0)^†^	51.3 (48.5–54.1)^†^	52.8 (48.9–56.6)^†^	48.4 (47.9–49.0)	49.4 (46.8–52.0)
Male	42.6 (41.9–43.4)	42.2 (41.5–43.0)	55.0 (50.8–59.3)^†^	42.0 (37.1–47.1)	45.3 (39.4–51.4)	NA	NA
Female	53.9 (53.2–54.6)	54.0 (53.2–54.7)	59.1 (53.9–64.2)^†^	54.9 (51.6–58.3)	57.8 (52.8–62.6)	NA	NA
**Confidence in vaccine safety (completely or very)**	64.4 (63.8–64.9)	63.9 (63.3–64.4)	76.3 (73.1–79.3)^†^	70.4 (67.6–73.1)^†^	69.3 (65.5–72.8)^†^	64.7 (64.2–65.2)	62.8 (60.2–65.4)
Male	64.1 (63.4–64.9)	63.2 (62.4–64.0)	82.4 (78.3–85.9)^†^	76.3 (71.0–80.8)^†^	72.7 (67.2–77.6)^†^	NA	NA
Female	64.6 (63.9–65.3)	64.5 (63.7–65.3)	67.4 (62.2–72.3)	68.1 (64.7–71.4)^†^	66.9 (61.8–71.7)	NA	NA
**Vaccine important to protect self (very or somewhat)**	80.9 (80.5–81.3)	80.4 (80.0–80.9)	90.8 (88.5–92.7)^†^	86.8 (84.7–88.6)^†^	83.5 (80.0–86.5)	80.7 (80.3–81.2)	83.2 (81.1–85.1)^§^
Male	77.7 (77.0–78.3)	76.9 (76.2–77.6)	92.9 (89.9–95.1)^†^	87.6 (82.7–91.2)^†^	80.8 (75.4–85.3)	NA	NA
Female	83.9 (83.4–84.5)	83.9 (83.2–84.4)	87.8 (83.9–90.9)^†^	86.4 (84.2–88.5)^†^	85.4 (80.5–89.2)	NA	NA
**Had friends/family who were vaccinated (almost all or many)**	70.2 (69.7–70.6)	70.0 (69.5–70.5)	76.4 (73.4–79.2)^†^	71.2 (68.4–73.8)	72.7 (69.1–76.0)	70.6 (70.1–71.1)	67.6 (65.1–70.1)^§^
Male	68.3 (67.6–69.0)	67.8 (67.1–68.6)	79.3 (75.5–82.8)^†^	71.5 (66.2–76.4)	71.4 (65.9–76.4)	NA	NA
Female	71.9 (71.3–72.6)	72.1 (71.4–72.8)	72.4 (67.4–76.9)	71.0 (67.7–74.1)	73.5 (68.7–77.8)	NA	NA
**Difficulty getting vaccinated (very or somewhat)^¶^**	14.9 (14.6–15.3)	14.9 (14.6–15.3)	18.9 (16.3–21.8)^†^	12.0 (10.4–13.8)^†^	16.1 (13.4–19.2)	15.1 (14.7–15.5)	13.2 (11.6–15.0)^§^
**Health care provider recommended the vaccine**	41.2 (40.7–41.7)	41.3 (40.8–41.9)	47.3 (44.1–50.6)^†^	38.1 (35.4–40.8)^†^	40.3 (36.7–44.1)	41.3 (40.8–41.8)	38.4 (35.9–40.9)^§^
Male	37.5 (36.8–38.2)	37.3 (36.5–38.0)	49.0 (44.7–53.3)^†^	34.2 (29.3–39.5)	37.2 (31.7–43.0)	NA	NA
Female	44.7 (44.0–45.4)	45.3 (44.6–46.1)	45.0 (40.0–50.1)	39.6 (36.4–42.8)^†^	42.5 (37.8–47.3)	NA	NA
**Work or school requires the vaccine**	22.6 (22.1–23.0)	22.1 (21.7–22.6)	26.4 (23.8–29.2)^†^	26.5 (24.2–29.0)^†^	29.4 (25.8–33.3)^†^	22.5 (22.1–23.0)	25.9 (23.6–28.2)^§^

**Abbreviation**: NA = not applicable.

* Weighted estimate.

^†^ Compared with heterosexual persons, p<0.05.

^§^ Compared with persons who were not transgender or nonbinary, p<0.05.

^¶^ Vaccinated respondents were asked, “How difficult was it for you to get a COVID-19 vaccine?”; unvaccinated respondents were asked “How difficult would it be for you to get a COVID-19 vaccine?

## Discussion

In this assessment of self-reported COVID-19 vaccination coverage and beliefs about COVID-19 vaccines among NIS-ACM survey respondents, receipt of ≥1 COVID-19 vaccine dose and vaccine confidence were higher among gay or lesbian adults than among heterosexual adults. In another large U.S. survey conducted during May–June 2021, 92% of LGBT respondents reported receiving ≥1 dose of a COVID-19 vaccine (*4*), but non-LGBT persons were not included; therefore, comparisons between LGBT and non-LGBT populations could not be made. In this assessment, disparities were also noted across subpopulations. Regardless of race or ethnicity, bisexual women were more confident in vaccine safety than were heterosexual women, and a higher percentage of gay or lesbian and bisexual women compared with heterosexual women thought COVID-19 vaccine was important to protect oneself. However, vaccination coverage was lower among non-Hispanic Black gay or lesbian and bisexual women than among non-Hispanic Black heterosexual women. Increasing availability of education about COVID-19 vaccine in local communities of color that promotes the benefits of vaccinations and provide opportunities to answer questions and receive COVID-19 vaccine might increase coverage among gay or lesbian and bisexual women.

With a higher prevalence of comorbidities that increase the risk for severe COVID-19 illness (*1*) LGBT persons might be at disproportionate risk for COVID-19 illness. Although awareness of these risks and disparities is essential for public health intervention, data on these populations are currently not widely available. Only two federally funded national surveys collect data on both sexual orientation and gender identity (*5*), with eight federally funded national surveys only collecting data on sexual orientation (*6*). Inclusion of sexual orientation and gender identity in surveys, as well as in COVID-19 testing, case reporting, and vaccination administration systems, can guide strategies to improve access to health care and prevention services among LGBT populations. This information could be used at the local level to reduce disparities in vaccination coverage among persons at highest risk for severe COVID-19–associated illness, such as non-Hispanic Black and Hispanic LGBT persons (*1*).

In addition to better characterizing the demographic characteristics of infected persons, information on sexual orientation and gender identity could potentially aid in response activities in the event of a COVID-19 outbreak among vaccinated persons. One such outbreak occurred in Barnstable County, Massachusetts in July 2021, which has a large population of LGBT residents and visitors, in which 74.0% of infections were among persons who were fully vaccinated and had traveled from 22 other states (*7*). Therefore, if the impact of an outbreak among LGBT persons is known, messaging to the LGBT community at the national level could help enhance appropriate test seeking or health care.

The findings in this report are subject to at least six limitations. First, NIS-ACM had a low response rate (20.9% in both September and October 2021), which can increase the potential for bias if systematic differences exist between respondents and nonrespondents, even after adjusting for nonresponse. Second, COVID-19 vaccination was self-reported and is therefore subject to recall or social desirability bias. Third, receipt of ≥1 doses of COVID-19 vaccine was assessed versus receipt of all recommended COVID-19 vaccinations. Fourth, survey respondents might not have identified as one of the sexual orientation categories provided or as transgender or nonbinary, and therefore might not have selected lesbian, gay, bisexual, or transgender or nonbinary. Fifth, the survey excluded institutionalized persons and those with no access to cellular telephones; however, 97.0% of Americans own some type of cellular phone (*8*). Finally, smaller sample sizes of LGBT persons might have yielded low statistical power to detect differences by sexual orientation and gender identity in stratified analyses. To mitigate possible bias, survey weights were calibrated to COVID-19 vaccine administration data by age group and sex within jurisdictions.

To prevent serious illness, hospitalization, and death, which are more common in unvaccinated persons than in those who have been vaccinated (*9*), it is important that all persons in the United States, including those in the LGBT community, stay up to date with recommended COVID-19 vaccinations. Understanding COVID-19 vaccination coverage and confidence among LGBT populations, and identifying the conditions under which disparities exist, can help tailor local efforts to increase vaccination coverage. Identifying drivers of vaccine acceptance in populations with high vaccine coverage, such as non-Hispanic White gay men, or drivers of vaccine hesitancy in populations with low vaccine coverage, such as non-Hispanic Black gay or lesbian women, could guide strategies to increase coverage among populations with lower vaccination coverage. Adding sexual orientation and gender identity to national data collection systems would be a major step toward monitoring disparities and developing a better-informed public health strategy to achieve health equity for the LGBT population.

**Summary**

**What is already known about this topic?**

Lesbian, gay, bisexual, and transgender (LGBT) persons are at increased risk for severe COVID-19 illness because of a higher prevalence of comorbidities.

**What is added by this report?**

COVID-19 vaccination coverage and vaccine confidence were higher among gay or lesbian adults than among heterosexual adults and higher among gay men than gay or lesbian women. There were no significant differences in vaccination coverage among persons based on gender identity. Vaccination coverage was lowest among non-Hispanic Black LGBT persons across all categories of sexual orientation and gender identity.

**What are the implications for public health practice?**

To prevent serious illness and death, all persons in the United States, including those in the LGBT community, should stay up to date with recommended COVID-19 vaccinations.

## Footnotes

* Vaccination was defined as receipt of ≥1 dose of BNT162b2 (Pfizer-BioNTech), mRNA-1273 (Moderna), or Other COVID-19 vaccine, or 1 dose of Ad.26.COV2.S (Janssen [Johnson & Johnson]) COVID-19 vaccine.

† Survey weights were calibrated to match the number of persons reported in each jurisdiction by sex and age group as of mid-month, as reported by jurisdictions to CDC.

§ Response rate was calculated according to the American Association for Public Opinion Research type 3 response rate. https://www.aapor.org/AAPOR_Main/media/publications/Standard-Definitions20169theditionfinal.pdf

¶ Survey respondents were asked a series of questions on perceived COVID-19 risk, current COVID-19 vaccination status, and attitudes and perceived barriers to getting vaccinated (https://www.cdc.gov/vaccines/imz-managers/nis/downloads/NIS-ACM-Questionnaire-Q2-2021_508.pdf). These questions are based on the Behavioral and Social Drivers framework for increasing vaccine confidence. https://www.cdc.gov/vaccines/covid-19/downloads/vaccination-strategies.pdf.

** 45 C.F.R. part 46.102(l)(2), 21 C.F.R. part 56; 42 U.S.C. Sect. 241(d); 5 U.S.C. Sect. 552a; 44 U.S.C. Sect. 3501 et seq.

†† Urbanicity status was derived based on the centroid of the zip code of residence, categorized as MSA principal city, MSA nonprincipal city, or non-MSA. An MSA is defined by the U.S. Office of Management and Budget (OMB) as consisting of “at least one urbanized area of 50,000 or more population, plus adjacent territory that has a high degree of social and economic integration with the core as measured by commuting ties.” MSAs are geographically delineated by groupings of neighboring counties and can cross state boundaries; names are assigned by OMB based on the names of one to three principal cities or places within each MSA. https://www.census.gov/topics/housing/housing-patterns/about/core-based-statistical-areas.html
